# Spotting lesions in thorax X-rays at a glance: holistic processing in radiology

**DOI:** 10.1186/s41235-022-00449-8

**Published:** 2022-11-22

**Authors:** Merim Bilalić, Thomas Grottenthaler, Thomas Nägele, Tobias Lindig

**Affiliations:** 1 Department of Psychology, University of Northumbria at Newcastle, Ellison Building, Newcastle upon Tyne, NE1 8ST UK; 2 Department of Neuroradiology, University of Tübingen, Tübingen, Germany

**Keywords:** Expertise, Radiology, Holistic processing, Inversion effect, Global impression

## Abstract

Radiologists often need only a glance to grasp the essence of complex medical images. Here, we use paradigms and manipulations from perceptual learning and expertise fields to elicit mechanisms and limits of holistic processing in radiological expertise. In the first experiment, radiologists were significantly better at categorizing thorax X-rays when they were presented for 200 ms in an upright orientation than when they were presented upside-down. Medical students, in contrast, were guessing in both situations. When the presentation time was increased to 500 ms, allowing for a couple more glances, the radiologists improved their performance on the upright stimuli, but remained at the same level on the inverted presentation. The second experiment circumvented the holistic processing by immediately cueing a tissue within the X-rays, which may or may not contain a nodule. Radiologists were again better than medical students at recognizing whether the cued tissue was a nodule, but this time neither the inverted presentation nor additional time affected their performance. Our study demonstrates that holistic processing is most likely a continuous recurring process which is just as susceptible to the inversion effect as in other expertise domains. More importantly, our study also indicates that holistic-like processing readily occurs in complex stimuli (e.g., whole thorax X-rays) but is more difficult to find in uniform single parts of such stimuli (e.g., nodules).

## Introduction

Medical images carry a wealth of information. Finding a potentially life-threatening abnormality within them is a difficult task that takes time and requires extensive training. Despite this, experienced radiologists often need only a glance to establish that something is amiss with the image. Radiologists rely on holistic processing, where complex stimuli are perceived as whole instead a collection of individual elements, to quickly identify relevant aspects of the medical image. This ability to quickly extract a global impression of the image (Krupinski, 2010) is assumed to be at the heart of radiological expertise. Here, we demonstrate that experienced radiologists do indeed need just a split second to grasp the essence of a radiological image. The initial global impression is improved with further exposure, but the performance remained impaired when the stimuli were presented in an unnatural manner, upside-down, regardless of the additional time. Most importantly, we identify situations where this holistic-like processing readily occurs, such as complex stimuli (e.g., whole thorax X-rays), and where it plays little part, such as isolated uniform parts of such stimuli (e.g., nodules).

### Theories of radiological expertise

One of the most convincing demonstrations of the global impression phenomenon has been provided by so-called flash experiments, where the stimuli are presented for such a brief period that a deliberate search is precluded. For example, in a seminal study, Kundel and Nodine (1975) demonstrated that experienced radiologists noticed more than 70% of abnormalities within a 200 ms presentation of X-rays (or *d*′ = 1 where *d*′ = 0 is a chance level). More recently, several studies (Brennan et al., 2018; Carrigan et al., 2018, 2019; Evans et al., 2013, 2016) demonstrated similar findings in mammography, where exposure of 250 ms or 500 ms was enough for above-chance detection of abnormalities in mammograms (*d*′ ≈ 1).

Even when the abnormalities were not noticed immediately, that is when there is a subsequent search, the global impression may power radiologists’ highly efficient search process. Unlike inexperienced colleagues who need to examine most of the image to locate the lesion, radiologists quickly fixate the problematic area (Carmody et al., 1981; Donovan & Litchfield, 2013; Wood et al., 2013). For example, Kundel et al. (2008) demonstrated that radiologists could fixate on two-thirds of cancers already within the first second of viewing a mammogram. The underlying assumption is that the initial global impressions guide radiologists’ attention toward more important aspects (e.g., abnormalities) of the stimulus and away from the irrelevant ones (Sheridan & Reingold, 2017). The remarkable ability of radiologists to quickly extract a gist of an image is confined to stimuli from their specialization. When other general stimuli (e.g., scenes, objects) are used in similar tasks, radiologists perform no better than people with little or no radiological training (Bilalić et al., 2016; Litchfield & Donovan, 2016; Nodine & Krupinski, 1998).

The importance of the first impression and its subsequent influence on the search phase is evident in theories of radiological expertise. In Swensson’s (1980) Two-Stage Detection Model, experts first use their superior perceptual skills to filter images for possible clues about abnormalities. The initial impression then drives the second stage, where identified locations are searched more thoroughly. In Nodine and Kundel’s (1987) Global-Focal Search Model, the initial impression (global search) of the image is compared to previously stored knowledge for possible abnormalities, which then leads to focusing attention on locations which may contain abnormalities (focal search). In the latest iteration of the model, named the Holistic Model (Kundel et al., 2007), holistic processing enables expert radiologists to quickly grasp the essence of the image, which again leads to a highly selective subsequent search for abnormalities as the search is based on the information extracted from the initial gestalt.

While there are differences between these models, most notably regarding whether they assume that the initial (global impression) and subsequent phases (search) are serial or parallel in nature (for a review, see Sheridan & Reingold, 2017), they all assume that radiologists have acquired an impressive wealth of knowledge about radiological images due to extensive previous exposure to visual images within their specialization. This knowledge, stored in long-term memory (LTM), represents an extensive repertoire of visual patterns, which include recurring visual features in radiological images. These visual patterns are meaningful units, chunks of information (e.g., the typical appearance of pneumonia and its variations), which are acquired, refined, and extended through practice and experience. The stored knowledge, in the form of chunks of domain-specific information, is then compared with the incoming information (Nodine & Mello-Thoms, 2000; Reingold & Sheridan, 2011; Sheridan & Reingold, 2017). Most experienced experts possess highly developed knowledge which enables them to quickly obtain a global impression of the image. A consequence of this initial phase of the radiological process is a selective search where experienced radiologists tend to focus on the important aspects of the stimuli (Sheridan & Reingold, 2017). Once a potential abnormality has been identified through the search guided by global impression, the final phase is recognition of the potential abnormality (Kundel et al., 2007).

A series of recent studies have proposed another distinctive mechanism based on the initial gist. Radiologists regularly notice the presence of abnormalities in briefly presented medical images, but unlike in the above-described research, they are unable to point out the areas where the lesions are located (Evans et al., 2013, 2016). They can, however, correctly categorize the stimulus as abnormal when they have only seen the healthy half of the otherwise abnormal stimulus (Evans et al., 2016). Even more astonishingly, this initial general suspicion can be diagnostic of abnormalities that will only occur in the future (Evans et al., 2019). This ‘global gist for abnormalities’ seems to be driven by the global image structure, which can be extracted quickly, rather than by the density or asymmetry of the image (Evans et al., 2016). Unlike the postulated global impression during the holistic processing, which is supposed to improve with additional exposure to the stimuli (Kundel & Nodine, 1975; Oestmann et al., 1993), the global gist for abnormalities seems to be static. Additional time, including unlimited viewing time, does not improve on the initial hunch about the presence of abnormalities in medical images (Gandomkar et al., 2021; Raat et al., 2021).

### Perceptual learning and visual perception

While the field of radiological science has mostly developed independently of that of cognitive science (Reingold & Sheridan, 2011), the influence of cognitive theories on radiological theories is nevertheless evident. For example, the Two-stage Detection Model (Swensson, 1980) was embedded in the framework of the signal detection theory (Green & Swets, 1966). The research on global gist (Evans et al., 2016) draws on the two-pathway model of scene perception (Wolfe et al., 2011) where it is also possible to identify the presence of an object without knowing its identity or location through the non-selective pathway (Drew et al., 2013; Evans et al., 2011). Finally, the phenomenon of global impression from the Two-stage Detection Model (Swensson, 1980) and Global-Focal Search Model (Nodine & Krupinski, 1998) has been equated in its latest iteration (Holistic Model; Kundel et al., 2008) with the holistic processing featured in the perceptual learning field (Richler et al., 2012).

The research on holistic processing in perceptual learning is important, because, not unlike in radiology, it indicates that in the process of acquiring expertise, there is a developmental shift. Namely, a featural, part-based perceptual strategy, where individual characteristics of objects are inspected to determine their category, is replaced by holistic processing, where the whole stimulus is perceived as a gestalt (Richler et al., 2012). The exact mechanisms of holistic processing are under considerable debate, but most of the proposals involve the perception of individual parts and the relations between them (Gauthier & Tarr, 2016). Arguably the most impressive example of holistic processing is face perception (Bartlett & Searcy, 1993). People are highly efficient at processing faces as they do not need to pay attention to individual parts but rather perceive faces as a whole. However, when faces are presented inverted (upside-down), their perception is considerably less efficient—the inversion effect (Yin, 1969). It had previously been assumed that only faces elicit holistic processing, either because of their special importance in human life or because of people’s immense practice and experience with faces (McKone et al., 2007). Recent evidence suggests, however, that holistic processing is a general characteristic of expertise (Bilalić, 2016; Bilalić et al., 2011a, 2011b, 2011c; Burns et al., 2019; Busey & Vanderkolk, 2005; Gauthier & Tarr, 1997; Vogelsang et al., 2017).

The inversion effect has also been demonstrated in radiology. Oestmann (1993) tested three experienced radiographers on the detection of abnormalities in briefly presented X-rays (250 ms) in upright and inverted positions. The performance of the radiology readers was consistently better when the stimuli were presented in the usual upright orientation than when inverted, regardless of whether obvious or subtle pathologies were present in the X-rays. The radiographers’ performance at first increased with additional time for the upright presentation (going from *d*′ ≈ 0.8 in 250 ms to *d*′ ≈ 1 in 1000 ms) but remained similar with extra additional time (four seconds or unlimited time). The inverted presentation, however, still produced above-chance detection at the 250 ms presentation (*d*′ between 0.45 and 0.75). Most surprisingly, the increased presentation time yielded the same pattern of initial increase and diminishing returns in the inverted orientation as in the common upright presentation. This indicates that a similar process is taking place on inverted stimuli, only that it is somewhat delayed.

Recently, Chin et al. (2018) extended these results by using not only an additional control task, but also a control group which was lacking in Oestmann’s study. Both experienced radiologists and novice radiology residents displayed the inversion effect when they had to detect emotions in faces presented for 250 ms (the difference between upright and inverted faces, Δd′ ≈ 1). Experienced radiologists, however, were not only much better in detecting abnormalities in briefly presented mammograms, but also displayed the inversion effect (Δd′ ≈ 0.3), unlike the novice residents. Unfortunately, the study used only the 250 ms presentation, which precluded checking the influence of additional time on the detection of abnormalities in upright and inverted radiological images.

### (General) Expertise

General theories of expertise (Bilalić, 2017; Chase & Simon, 1973; Gobet & Simon, 1996b) also postulate similar processes as in radiology (see, also Wood, 1999). The key is, again, the acquired knowledge structures which feature numerous individual elements and their relations. They enable experts to recognize patterns in the incoming stimuli and automatically retrieve ways of dealing with new situations (Gobet et al., 2001). The allocated attention based on the initial pattern recognition then draws focus on important aspects, which in turn feed back to and refine the initially recognized patterns (Bilalić, 2017; Chase & Simon, 1973; Gobet & Simon, 1996b). Chess experts, for example, can quickly grasp the essence of a normal chess position and immediately come up with promising solutions (Bilalić et al., 2008; de Groot, 1978). The examination of those solutions will inevitably trigger further patterns, which may or may not refine the initial mental model and lead to the revision of the initial solution (Bilalić, 2017; Chase & Simon, 1973; Gobet & Simon, 1996b). Similarly, tennis players may initially use the position of the opponent’s feet and knees to get an idea about the direction and power of a tennis serve, but that initial guess will constrain further perception of other relevant body parts, such as shoulders and the serving hand (Williams & Jackson, 2019). Regardless of the nature of knowledge, whether it is perceptual as in radiology, cognitive as in chess, or kinetic as is the case in sport, the universal expertise mechanism assumes that an initial guess based on pattern recognition is continuously refined by the attentional–perceptual loop it has caused (Bilalić, 2017).

One way to illustrate the expertise mechanism is to randomly distribute elements in the environment and therefore break the meaningful relations between them. Once there are no meaningful patterns in the environment to connect with existing structures in the memory, the performance suffers (Bilalić et al., 2010, 2011a, 2011b, 2011c; Chase & Simon, 1973; Gobet & Simon, 1996a). This is evident in tasks that do not feature multiple elements and therefore do not necessarily require chunking processes. Chess experts are still faster than chess novices when they deal with two or three isolated objects (e.g., there is a check relation between pieces), but their advantage is considerably smaller than when they deal with normal positions which feature numerous objects and relations between them (Bilalić, 2016; Bilalić et al., 2011a, 2011b, 2011c). The expert advantage becomes even smaller, and sometimes not significant, when the task is to recognize a single object, especially if there is no need to locate the object first (Bilalić, 2016). Here, experts rely, not on the complex pattern recognition required in the multi-relation environment, but rather on familiarity with the individual stimuli and use of parafoveal vision (Bilalić et al., 2011a, 2011b, 2011c; Reingold et al., 2001a, 2001b; Reingold et al., 2001a, 2001b).

### Current study

**Fig. 1:**
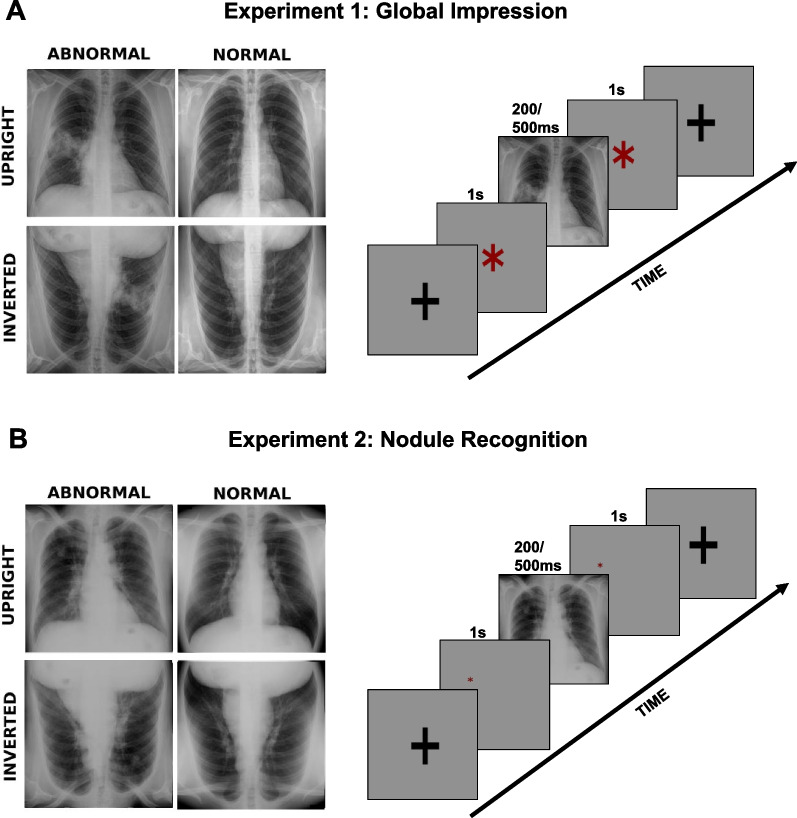
Design and Stimuli. **A** In the first experiment (global impression), radiologists and medical students were presented with thorax X-rays with lesions (abnormal) and without lesions (normal) in an upright and inverted orientation for 200 ms or 500 ms. Before the actual presentation of X-rays, a screen lasting for 1000 ms was presented, preparing the participants for the upcoming stimulus. After the X-ray had disappeared, another screen was presented where the participants had to indicate their response. **B** In the second experiment (nodule recognition), radiologists and medical students were presented with thorax X-rays in an upright and inverted orientation for 200 ms or 500 ms. Before the actual presentation of X-rays, a preparation screen with the cue where the supposed nodule would appear was shown for 1000 ms. After the X-ray had disappeared, another screen was presented where the participants had to decide whether the tissue presented contained a nodule

Here, we elucidate the mechanisms and limits of holistic processing in radiology by using typical manipulations from the fields of perceptual learning and expertise. In the first experiment (Fig. 1A), we combine the flash presentation with the inversion manipulation. We present the radiological images for a mere 200 ms, which essentially only allows for a glance, in upright and inverted positions. We expect to replicate the previous findings of experts’ superiority and the inversion effect (Bilalić et al., 2016; Chin et al., 2018; Oestmann et al., 1993). We go further than the previous research; however, in that we also include a condition where the radiological images were presented for 500 ms, enough time to allow for an additional glance or two. In accordance with the theories of radiological (Kundel et al., 2007, 2008) and (universal) expertise (Bilalić, 2017), we expect that expert radiologists will be able to improve upon their initial global impression when given additional time (Oestmann et al., 1988, 1993). However, if the inversion effect disturbs the holistic processing, we would not expect that the additional 300 ms would be enough to offset the lack of familiarity with the inverted presentation even if the same holistic processing is only delayed (see, Richler et al., 2011).

Our second experiment sets out to establish the limits of holistic processing in radiology, or in other words, instances where holistic processing is absent (Kuhn, 1970; Popper, 1957; Wason, 1960). Inspired by similar paradigms in the expertise field (Bilalić, 2017; Gobet et al., 2004), we circumvent holistic processing by directly cueing small parts of tissue within the image and ask whether they contain nodules (for similar albeit implicit manipulations in radiology, Carrigan et al., 2019). We want to demonstrate that the recognition of individual lesions, in contrast to the processing of the whole stimulus, may not be driven by holistic processing to the same extent. Experts should be better at recognizing individual nodules, but their advantage over novices should be considerably smaller than in the first experiment when holistic processing was required (Bilalić, 2016; Bilalić et al., 2011a, 2011b, 2011c). Most importantly, if the holistic processing is irrelevant, the inversion manipulation should not influence experts’ performance, unlike in the first experiment.

## Experiment 1: global impression

### Method

#### Participants

There were 16 radiologists (6 female, *M* age ± *SD* age = 35.2 ± 4.3) and 18 medical students (6 female, *M* age ± *SD* age = 28.2 ± 4.7) at Tübingen University. The radiologists, who were all recruited from the Department of Radiology at the University Hospital Tübingen, had over 5 years of experience and had examined over 10,000 X-rays. The medical students were recruited from the final (sixth) year of the basic medical course at the University Hospital Tübingen and had limited (course- and textbook-related) experience with X-rays. The Ethics Committee of Tübingen University approved the study. All participants signed informed consent, and the study was performed in accordance with ethical standards outlined by the Declaration of Helsinki.

#### Task and design

In the first experiment (Global Impression), the participants were asked to indicate whether the presented thorax X-ray image contained an abnormality (Fig. 1A). The X-rays were presented for either 200 ms or 500 ms in upright or inverted (‘flipped vertically’) orientation. We first presented the X-ray trials lasting 500 ms and then those lasting 200 ms. This was done because our pilot studies showed that the participants had more difficulties when they dealt immediately with the fast condition, 200 ms presentation time, than when they experienced the slower condition, 500 ms, first. More specifically, the time needed to get accustomed to the uncommon presentation speed was much larger with the 200 ms presentation. This had unwanted consequences on the duration of the experiment (as well as on the moral of participants). The upright and inverted X-rays were presented randomly within a single condition (e.g., within 200 and 500 ms duration).

The stimuli were presented to all participants in an isolated room (located at the Department of Radiology at the University Hospital Tübingen) on a 32″ screen connected to a computer, which used Presentation® software (Version 16.0, Neurobehavioral Systems, Inc., Berkeley, CA, www.neurobs.com) for running the experiments. The screen was around one meter away from the participants.

#### Stimuli

The X-rays in the first experiment were taken from the internal database of the department of radiology at the medical clinic of Tübingen University, a freely available digital image database (Shiraishi et al., 2000), and from a recent study (Melo et al., 2011). There were altogether 98 (unique) X-ray images, 48 in each of the two duration conditions. Different images were used in both conditions. Out of 48 images in one condition, 24 were presented in the upright orientation, and 24 inverted. Half of those 24 images were abnormal X-rays with a lesion, and the other half were normal images without lesions. The lesions ranged from small (e.g., atelectasis), to medium (e.g., bulla), to large (e.g., tuberculosis, pneumonia). There were eight examples/images in each of the three size categories. The location of lesions (i.e., left/right lung field) was counterbalanced, and all 48 images were presented randomly for each participant (i.e., there were no blocks of certain kinds of stimuli). All stimuli had the same dimensions of 1024 × 1024 pixels. The stimuli subtended ∼ 17° visual angle with the targets in the abnormal stimuli subtending ∼ 2°.

#### Data analysis

The performance was measured by the sensitivity index *d*′, which was obtained by calculating the normal-transformed hit rate (the proportion of times each participant said “abnormal” when the presented X-ray contained an abnormality, in the first experiment, or the nodule was cued, in the second) and subtracting from it the normal-transformed false alarm rate (the proportion of times each participant said “abnormal” when the presented X-ray did not feature abnormalities or the cued tissue was a nodule). We first checked whether the participants were guessing by comparing their accuracy *d*′ against chance level (*d*′ = 0) using one-sample *t* tests. In Experiment 1, we used the analysis of variance (ANOVA) with three factors: expertise (expert/novice), time (200/500 ms), and orientation (upright/inverted). In some cases, we used paired *t* tests to examine the differences between conditions among experts and novices. Cohen’s *d* was used as the measure of effect size for t tests, which represents the difference between the two means in standard deviation units. *d* = 0.2 is considered a small effect size, 0.5 a medium one, and 0.8 a large one (Cohen, 1977). For ANOVAs, we used partial eta-squared, $\eta_{p}^{2}$, which measures the proportion of the total variance in a dependent variable that is associated with an independent variable when other independent variables and interactions are partialed out. In that sense, it is generally larger than the percentage of explained variance (coefficient of determination). $\eta_{p}^{2}$ < 0.10 is considered a small effect, $\eta_{p}^{2}$ < 0.25 a medium effect, and 0.40 a large effect (Cohen, 1977).

### Results and discussion

**Fig. 2:**
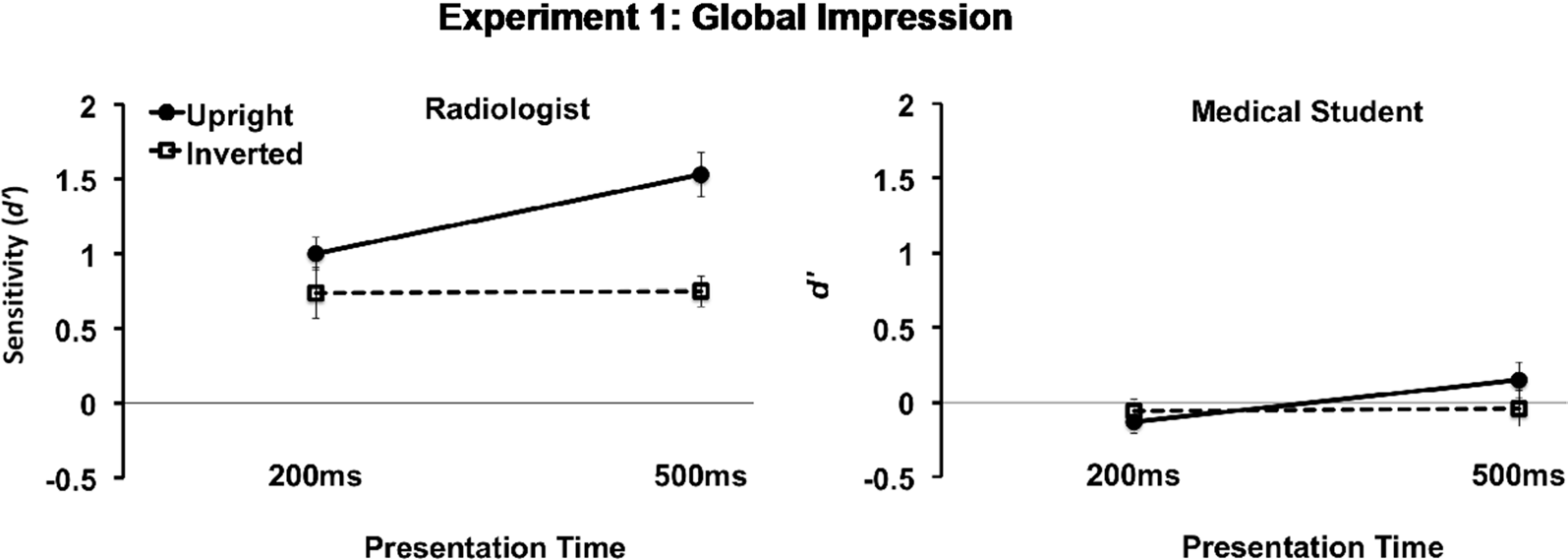
Experiment 1 (Global Impression) results. Performance, as measured by sensitivity *d*′, on the global impression task for radiologists (left) and medical students (right) on upright and inverted X-rays presented for 200 ms and 500 ms

Figure 2 shows that radiologists performed at a high level as measured by the sensitivity index *d'* and were significantly better than chance in classifying the images in the 200 ms condition (e.g., for upright X-rays, *t*(15) = 9.4, *p* < 0.001; for inverted *t*(15) = 4.3, *p* = 0.001). Medical students, on the other hand, were guessing, as their accuracy level was not significantly better than chance (for upright *t*(17) = − 0.2, *p* = 0.87; for inverted *t*(17) = − 0.8, *p* = 0.45). The same pattern of results was found when the X-rays were presented for 500 ms: experts were above the chance level (upright *t*(15) = 9.9, *p* < 0.001; inverted *t*(15) = 7.5, *p* < 0.001) whereas novices were guessing, although their performance with the upright X-rays displayed trends of non-guessing performance (upright *t*(17) = 1.2, *p* = 0.23; inverted *t*(17) =  − 0.3, *p* = 0.76).

Three-way ANOVA with expertise (expert/novice), time (200/500 ms), and orientation (upright/inverted) indicated that radiologists were more accurate than medical students overall (main effect of expertise: *F*1,32 = 59.5, MSE = 33.6, *p* < 0.001, $\eta_{p}^{2}$ = 0.65). Both groups profited from longer presentation time (main effect of Time: *F*1,32 = 10.6, MSE = 1.1, *p* = 0.003, $\eta_{p}^{2}$ = 0.25) to a similar effect (interaction Expertise × Time was not significant: *F*1,32 = 2.5, MSE = 0.26, *p* = 0.12, $\eta_{p}^{2}$ = 0.07). Participants were also more accurate with upright than inverted presentation (main effect of Orientation: *F*1,32 = 22.1, MSE = 3.6, *p* < 0.001, $\eta_{p}^{2}$ = 0.41). However, only experts reaped the benefits of the common orientation of the X-rays, as novices’ performance did not differ greatly between the upright and inverted orientations (interaction Expertise × Orientation: *F*1,32 = 8.9, MSE = 1.4, *p* = 0.006, $\eta_{p}^{2}$ = 0.22).

Finally, the longer duration improved radiologists’ accuracy on upright but not on inverted X-rays (interaction Time × Orientation: *F*1,32 = 6.8, MSE = 0.9, *p* = 0.014, $\eta_{p}^{2}$ = 0.18). Even the three-way interaction Expertise × Time × Orientation was not significant (*F*1,32 = 2.3, MSE = 0.3, *p* = 0.14, $\eta_{p}^{2}$ = 0.07); the benefit of additional time for upright orientation was pronounced in experts (two-way interaction Time × Orientation: *F*1,15 = 14.1, MSE = 2.9, *p* = 0.002, $\eta_{p}^{2}$ = 0.48; paired *t* test between upright in 500 ms and 200 ms *t*(15) = 4.5, *p* > 0.001 Cohen’s *d’* = 0.94) but was not significant in novices (*F*1,16 = 1.3, MSE = 0.4, *p* = 0.27, $\eta_{p}^{2}$ = 0.08; paired *t*(17) = 1.5, *p* = 0.15, *d*′ = 0.36).

## Experiment 2: nodule recognition

### Method

#### Participants

The same participants as in the previous experiment took part, except for one medical student.

#### Task and design

In the second experiment (Nodule Recognition), the participants were asked to indicate whether the cued tissue within a thorax X-ray image contained a nodule (Fig. 1B). The X-rays, which either contained nodules or did not contain nodules, were presented for either 200 ms or 500 ms in an upright or inverted position. Before presenting the image, a warning image containing a small red star at the place where the nodule would eventually appear was presented for one second. Otherwise, the same setup as in Experiment 1 was used.

#### Stimuli

The X-rays in the second experiment were taken from the freely available digital image database of the Japanese Society of Radiological Technology (JSRT: Shiraishi et al., 2000; http://db.jsrt.or.jp/eng.php). The nodules in the database were established as malignant based on histologic and cytologic examination and classified into five difficulty categories based on the performance of 20 radiologists. In the condition with nodules, only one nodule is present in each X-ray. The nodule location was counterbalanced across lung fields. The JSRT database also featured X-rays without nodules. These X-rays were used for the condition without nodules. Since the X-rays had no nodules, location cues were placed in highly similar locations as in the X-rays with nodules (i.e., at least the same quadrant). An experienced radiologist (the senior author) checked the cue locations for plausibility (i.e., location and whether the tissue could contain nodule). The whole stimulus subtended ∼ 17° visual angle with nodules subtending ∼ 1°.

#### Data analysis

The data analysis for Experiment 2 followed the same pattern as in Experiment 1. We used a three-way ANOVA (expertise × time × orientation) on the performance measure *d'*.

## Results and discussion

**Fig. 3:**
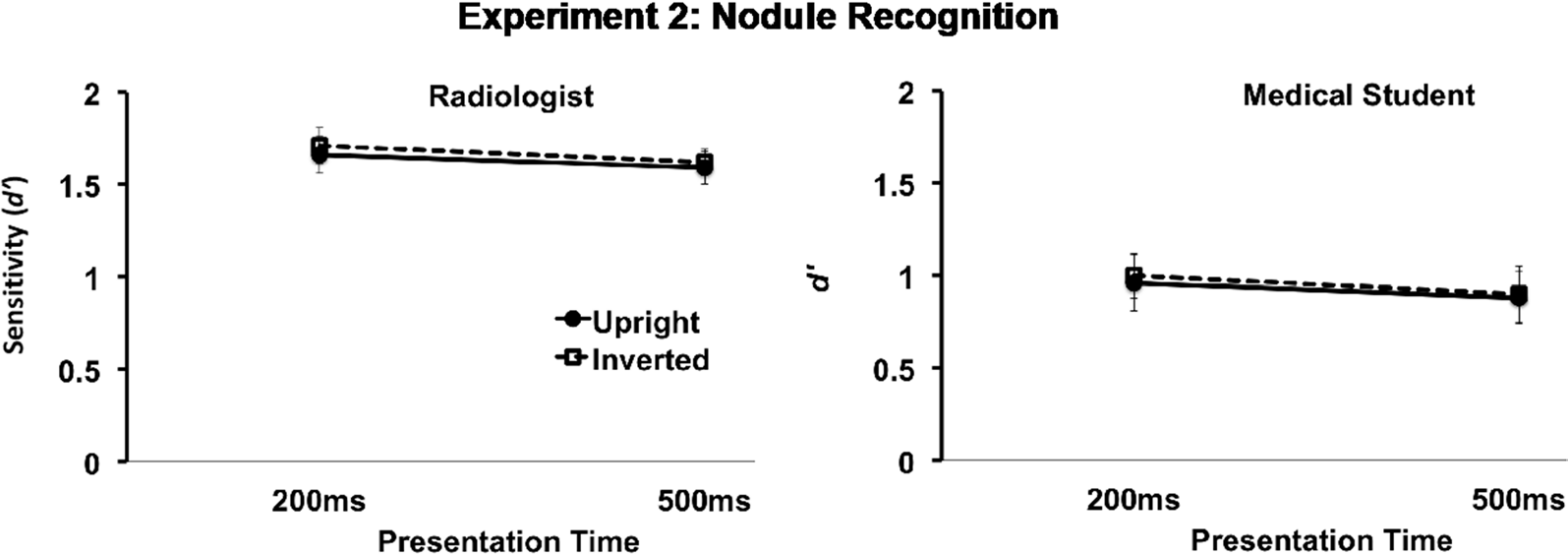
Experiment 2 (Nodule Recognition) results. Performance, as measured by sensitivity *d*′, on the nodule recognition task for radiologists (left) and medical students (right) on upright and inverted X-rays presented for 200 ms and 500 ms

Figure 3 shows that radiologists were above the chance level in deciding whether or not the nodules were present in both orientations and both time durations (200 ms: upright *t*(15) = 17.2, *p* < 0.001 and inverted *t*(15) = 16.4, *p* < 0.001; 500 ms: upright *t*(15) = 17.2, *p* < 0.001 and inverted *t*(15) = 16.4, *p* < 0.001). However, unlike in the global impression task in the previous experiment, the medical students were also performing above the chance level when it came to recognizing nodules (200 ms: upright *t*(16) = 6.5, *p* < 0.001 and inverted *t*(16) = 8, *p* < 0.001; 500 ms: upright *t*(16) = 6.2, *p* < 0.001 and inverted *t*(16) = 6, *p* < 0.001). Nevertheless, radiologists were more accurate overall in nodule recognition than medical students (main effect of Expertise: *F*1,31 = 25.7, MSE = 16.7, *p* < 0.001, $\eta_{p}^{2}$ = 0.45).

Unlike in the previous experiment on global impression, the longer duration was not beneficial for performance (main effect of Time *F*1,31 = 1.5, MSE = 0.24, *p* = 0.23, $\eta_{p}^{2}$ = 0.05; interaction Expertise × Time *F*1,31 = 0.005, MSE = 0.001, *p* = 0.95, $\eta_{p}^{2}$ = 0.001). Similarly, the orientation of the X-rays had no effect (main effect Orientation: *F*1,31 = 0.5, MSE = 0.39, *p* = 0.47, $\eta_{p}^{2}$ = 0.02; interaction Expertise × Orient *F*1,31 = 0.04, MSE = 0.003, *p* = 0.85, $\eta_{p}^{2}$ = 0.001) nor was there any interaction between duration and orientation (*F*1,31 = 0.03, MSE = 0.002, *p* = 0.87, $\eta_{p}^{2}$ = 0.001). The three-way interaction between expertise, time, and orientation was also not significant (*F*1,31 = 0.001, MSE = 0.001, *p* = 0.98, $\eta_{p}^{2}$ = 0.001).

## General discussion

The radiologists were susceptible to the inversion effect, as in other perceptual expertise domains (Bilalić, 2016; Bilalić et al., 2011a, 2011b, 2011c; Burns et al., 2019; Busey & Vanderkolk, 2005; Gauthier & Tarr, 1997; Vogelsang et al., 2017). Radiologists categorized thorax X-rays above the chance level even when they were presented for a period that permitted only a single glance. This ability was significantly impaired when the X-rays were presented upside-down. The global impression is an acquired ability because medical students, who lack extensive training and knowledge about X-rays, were only guessing and consequently were not affected by the inversion effect. When the holistic processing is circumvented, as in the second experiment where the visually simple lesions are directly cued, the radiologists were still better than medical students, but the advantage was considerably smaller (Δ*d*′ ≈ 0.6) than in the first experiment where holistic processing was available (Δ*d*′ ≈ 1). Most importantly, there was no inversion effect present in either group.

### Holistic processing in radiology

Establishing the inversion effect in radiology provides another piece of evidence for the general nature of holistic processing (see also, Carrigan et al., 2019; Chin et al., 2018). Radiological images bear no visual resemblance to faces, unlike other stimuli for which holistic processing has been established (Kanwisher & Yovel, 2006). We can therefore be certain that the inversion effect in radiology is not a consequence of X-rays being similar in appearance to faces. Thorax X-rays, however, possess a conceptual similarity to faces because they are made of individual parts such as lungs and ribs that are always present at specific locations, like the eyes, nose, and mouth in faces. There are many variations of these individual parts, but experienced radiologists have experienced many such instances. Consequently, they are able to parse complex stimuli such as X-rays at a single glance, not unlike the way that most people can grasp faces within milliseconds.

Recent neuroimaging studies provide additional support for the role of holistic processing in radiological expertise. The fusiform face area (FFA), the same area that is held responsible for holistic processing of faces (Kanwisher et al., 1997) but also for the holistic processing of other stimuli (Chin et al., 2018; Gauthier et al., 2000), is found to differentiate between radiologists and medical students (Bilalić et al., 2016). More importantly, only the FFA in radiologists was able to discriminate between upright and inverted presented X-rays (Bilalić et al., 2016). Although this study did not test directly for holistic processing, it converges with other behavioral and neuroimaging evidence (Bilalić et al., 2016; Harley et al., 2009; Kok et al., 2021; Myles-Worsley et al., 1988) that establishes similarities between the processing of radiological images and that of faces.

We have demonstrated that only radiologists benefited from an additional presentation time of 300 ms, and only when the stimuli were presented in the normal upright orientation. We do not believe that the additional 300 ms, which would enable two or three further fixations at most, was sufficient to directly identify the lesions in the X-rays. Certainly, the radiologists in their post-experimental verbal protocols and debriefing rarely claimed that they directly spotted the lesions. We do believe, however, that the additional glances serve to improve and update the initial global impression. Radiologists move their eyes to a certain spot on the X-ray based on the initial global impression. It is reasonable to assume that they are then able to form a more precise global impression based on perceiving the surroundings. The updated global impression would then trigger another eye movement, making the radiological search process a combination of eye fixations based on the global impression.

This serial concept of updated initial global impression was first proposed in theories of expertise in which the recurring cycle of pattern recognition that powers the search process is at the core of experts’ performance (Bilalić, 2017; Bilalić et al., 2009; Chase & Simon, 1973; Gobet & Simon, 1996b, 1998). While the initial theories of radiological expertise assumed a rather static holistic process that occurs only at the initial stage (Swensson, 1980), similar dynamic explanations of holistic processing have been proposed in the radiological literature (for a review, see Sheridan & Reingold, 2017). For example, Nodine and Mello-Thomas (2000) speculated that global and focal phases could be serial in nature, forming a recurrent loop, while Kundel et al. (2007) proposed that both occur in parallel, simultaneously influencing each other. Comparable parallel mechanisms have been proposed for the interaction of selective and non-selective visual pathways in more recent theories of radiological expertise (Drew et al., 2013).

Assuming a constant, either serial or parallel updating of the initial global impression, instead of its fixed initial formation, may go a long way in explaining some surprising findings. Litchfield and Donovan (2016) reported a surprising finding that radiologists were not able to benefit from a flash preview of the medical image in their subsequent search. However, that search phase featured a highly restricted vision, as the radiologists were confined by a small gaze-contingent moving window in the search. It is therefore possible that this forced focus on isolated parts of the stimulus prevented radiologists from developing their understanding of the stimulus beyond the initial global impression.

### Inversion effect in radiology

The inversion hampered radiologists’ performance, but they were still able to extract enough information in the upside-down X-rays to categorize them above the chance level even when they were presented for only 200 ms. It is possible that the inverted orientation does not remove all the global impression processes in experts. Inverted faces may hamper general performance, but people are still able to deal with such stimuli when given enough time (Richler et al., 2011). Similarly, chess experts also perform better than novices when chess pieces are randomized on the chess board (Gobet & Simon, 1996a), the result that generalizes to other domains (Sala & Gobet, 2017). Chess experts are also considerably above the chance level in identifying the incongruent two halves of the chess position in the composite paradigm (Boggan et al., 2012).

In contrast to the upright stimuli, the performance on the inverted stimuli did not improve when the additional time was given. On the one hand, this is something one would expect given that the presentation of stimuli upside-down is supposed to impede holistic processing (Yin, 1969). On the other hand, the finding runs counter to the improvement found by Oestmann (1993). It is possible that the extra additional time provided in the Oestmann study (additional 800 ms vs. 300 ms here) was enough for holistic processes to take place. This explanation is somewhat consistent with the research on face perception, which indicates that holistic processing is merely delayed when the faces are presented upside-down. Whereas upright faces elicit holistic processing already at 180 ms, around 800 ms are necessary for the same processes to take place in the case of inverted faces (Richler et al., 2011). Another possibility is that the small number of participants in Oestmann’s study (i.e., merely three of them) was responsible for the surprising effect.

The differing effect of the additional time on upright and inverted stimuli may indicate that differing processes are responsible for the above-chance performance in these two conditions. It is possible that the holistic processing postulated by radiological theories (Kundel et al., 2007, 2008; Swensson, 1980) is responsible for the improvement with the upright stimuli. The additional 300 ms in the longer 500 ms condition enable a couple more glances, presumably guided by the initial global impression, which they would then further refine (Bilalić, 2017; Reingold & Sheridan, 2011; Sheridan & Reingold, 2017). This is not achievable with the inverted stimuli as the patterns of attentional allocation have been disrupted and the additional time is not enough to overcome the uncommon orientation. In contrast, the clearly above-chance performance of radiologists with the briefly presented inverted X-rays could be a consequence of the global gist for abnormalities. The research on gist abnormality has repeatedly shown that the unlocalized sense for the presence of abnormalities can be extracted in situations where there the signal is degraded or not even clearly visible, such as from healthy halves of the stimuli (Evans et al., 2016) or stimuli that are currently normal but will develop abnormalities in future (Evans et al., 2019). Similarly, the initial sense of abnormalities does not seem to improve with time, that is, it stays at the same level as it was with a flash presentation (Gandomkar et al., 2021; Raat et al., 2021). This is clearly a speculative assumption, but, to our knowledge, there are no studies on the global gist which featured the inversion effect, nor in which the inversion condition was combined with differing presentation time.

### Limits of holistic processing

Just as it is important to establish when holistic processing in radiology occurs, it is also important to find instances when it is absent (Kuhn, 1970; Popper, 1957; Wason, 1960). Our second experiment shows that when we circumvent the global impression and move directly to the final stage of recognition of pathologies, the inversion effect is absent. Radiologists are still vastly superior to medical students at recognizing nodules, but the performance remains constant even when the stimuli are shown upside-down. Manipulating the surrounding tissue has shown little effect on the behavioral performance of identifying nodules (Harley et al., 2009) and our experiment demonstrates that the direct change of nodules’ orientation has no effect either.

Unlike in the first experiment, which depended on parsing the relations of numerous individual parts, the recognition of nodules involved a small patch of tissue whose recognition was mostly unrelated to the surrounding tissue. The absence of functional and spatial relations between the individual parts of the processed stimuli gives rise to other, not necessarily holistic, processes. This has been indirectly confirmed by a series of neuroimaging studies on chess experts (for a review, see Bilalić, 2018). FFA in experts is more engaged than in novices when they are dealing with chess positions with multiple elements and their relations, regardless of the task (Bilalić et al., 2011a, 2011b, 2011c). When only one or two elements are presented in isolation, there is no difference in the FFA activation between experts and novices even if there are explicit relations (i.e., check) between these individual objects (Bilalić, 2016). Other areas important for global gestalt perception (Huberle & Karnath, 2011), such as temporo-parietal junction, also react only to complex multi-object stimuli and not to isolated individual objects (Rennig et al., 2013). Additional experiments with different lesions are necessary before it can be claimed that holistic processing does not play a significant role in the recognition phase of radiological expertise, but currently it seems that its influence is considerably diminished when isolated pathologies are examined.

The nodule recognition task was seemingly easier than the global impression task in Experiment 1. Medical students, for example, had above-chance performance when the cued stimuli were presented for only 200 ms. It is possible that the identification of nodules, due to their distinctive nature, is considerably simpler than in the case of other abnormalities. One should also keep in mind that our novices were medical students who had at least some training with X-rays and nodules, the categorization of which is part of their basic training. We can similarly only speculate why there was no improvement with the additional 300 ms for the inspection of the nodules. The performance was already relatively high in the 200 ms condition, and neither radiologists nor medical students could improve their performance. The high performance could be related to the simplicity of nodules, which tend to be uniform with clearly distinguished definitions. Nevertheless, radiologists achieved a comparable performance to the nodule identification (around *d*′ = 1.7) in a longer condition of the global impression task (*d*′ = 1.5).

### Limitations

There are several limitations to our study. The inversion effect is often indicative of holistic processing, but not always (Richler et al., 2011, 2012). Instead of reflecting the holistic processing of individual elements, the inversion effect could merely reflect sensitivity to orientation (Richler et al., 2012). A more direct test of holistic processing based on the failure of selective attention instead of orientation, such as the composite paradigm (Boggan et al., 2012; Farah et al., 1998), would be necessary.

Similarly, the design could have featured cueing abnormalities that are more complex visually, such as pneumonia and tuberculosis, instead of nodules in the second experiment. Unlike nodules, which tend to be uniform stimuli with clearly distinguished definitions, the abnormalities in this study (Experiment 1) tended to be less specified in addition to being larger, more complex stimuli. It is therefore possible that the inversion effect would be elicited with the same paradigm but with other, more visually complex stimuli than nodules. The same cueing paradigm with more visually complex lesions would further establish the circumstances where holistic processing breaks down.

It is tempting to assume that holistic processing (Kundel et al., 2007, 2008) and not global gist for abnormalities (Drew et al., 2013; Evans et al., 2013, 2016) is responsible for the improvement with the additional time in the upright condition (see Fig. 3, left panel). After all, it is known that the global gist is independent of the presentation time (Gandomkar et al., 2021; Raat et al., 2021). It is, however, difficult to exclude the possibility that both processes interact with each other to enable the performance, as we do not know whether the radiologists could indicate where exactly the lesions were present. In future, researchers may want to combine the additional checks for the location in their design with the upside-down presentation of stimuli in their study designs to disentangle the influence of holistic processing and the global gist in radiological expertise.

The medical students acted as a high-level control group as they come from the same population as radiologists. Their performance demonstrates that the performance of radiologists is acquired and not a product of some innate ability. Their performance in Experiment 1, however, is problematic as the task was too difficult for them even after extending the viewing time. More skilled novices, such as residents with a couple of years of experience, would probably be a more appropriate control group. Finally, we acknowledge that the number of participants may not be sufficient for detecting the interactions between the factors used in the studies. Although experts are by definition rare, and our sample is among larger ones in the expertise field, a larger sample of radiologists would be advantageous.

### Conclusion

Our study underlines the importance of holistic processing in radiology. It replicates the previous studies demonstrating the inversion effect (Bilalić et al., 2016; Chin et al., 2018; Oestmann et al., 1993), and it extends them by showing the dynamic recurrent nature of the holistic processing in radiological expertise (Kundel et al., 2007, 2008). While this finding is important for the debate on the nature of holistic processing, we also demonstrate the circumstances that are necessary for holistic processing to occur. The recognition of individual isolated lesions does not seem to be under the influence of a holistic-like processing.

## Data Availability

The data are available upon request from the corresponding author.
